# Incorporating patient-specific information for the development of rectal tumor auto-segmentation models for online adaptive magnetic resonance Image-guided radiotherapy

**DOI:** 10.1016/j.phro.2024.100648

**Published:** 2024-09-16

**Authors:** Chavelli M. Kensen, Rita Simões, Anja Betgen, Lisa Wiersema, Doenja M.J. Lambregts, Femke P. Peters, Corrie A.M. Marijnen, Uulke A. van der Heide, Tomas M. Janssen

**Affiliations:** a Department of Radiation Oncology, The Netherlands Cancer Institute, Plesmanlaan 121, 1066 CX Amsterdam, the Netherlands; b Department of Radiology, The Netherlands Cancer Institute, Plesmanlaan 121, 1066 CX Amsterdam, the Netherlands

**Keywords:** Rectal cancer, Deep learning, Auto-segmentation, GTV, MRI-guided radiotherapy, Online adaptive radiotherapy

## Abstract

**Background and purpose:**

In online adaptive magnetic resonance image (MRI)-guided radiotherapy (MRIgRT), manual contouring of rectal tumors on daily images is labor-intensive and time-consuming. Automation of this task is complex due to substantial variation in tumor shape and location between patients. The aim of this work was to investigate different approaches of propagating patient-specific prior information to the online adaptive treatment fractions to improve deep-learning based auto-segmentation of rectal tumors.

**Materials and methods:**

243 T2-weighted MRI scans of 49 rectal cancer patients treated on the 1.5T MR-Linear accelerator (MR-Linac) were utilized to train models to segment rectal tumors. As benchmark, an MRI_only auto-segmentation model was trained. Three approaches of including a patient-specific prior were studied: 1. include the segmentations of fraction 1 as extra input channel for the auto-segmentation of subsequent fractions, 2. fine-tuning of the MRI_only model to fraction 1 (PSF_1) and 3. fine-tuning of the MRI_only model on all earlier fractions (PSF_cumulative). Auto-segmentations were compared to the manual segmentation using geometric similarity metrics. Clinical impact was assessed by evaluating post-treatment target coverage.

**Results:**

All patient-specific methods outperformed the MRI_only segmentation approach. Median 95th percentile Hausdorff (95HD) were 22.0 (range: 6.1–76.6) mm for MRI_only segmentation, 9.9 (range: 2.5–38.2) mm for MRI+prior segmentation, 6.4 (range: 2.4–17.8) mm for PSF_1 and 4.8 (range: 1.7–26.9) mm for PSF_cumulative. PSF_cumulative was found to be superior to PSF_1 from fraction 4 onward (p = 0.014).

**Conclusion:**

Patient-specific fine-tuning of automatically segmented rectal tumors, using images and segmentations from all previous fractions, yields superior quality compared to other auto-segmentation approaches.

## 1 Introduction

Radiotherapy (RT) plays an important role in the management of rectal cancer [1]. Integrated Magnetic Resonance Image (MRI) linear accelerators (MR-Linac) [2] have enabled daily online adaptation on MRI with increased accuracy, using high soft tissue contrast MRI images to adapt daily plans to anatomical variations between treatment fractions. Furthermore, it enables real-time monitoring and adjustment to changes occurring within the treatment fraction [3].

However, online adaptation is time- and resource-intensive with manual re-contouring of the target volumes and organs at risk (OAR) at every treatment fraction, constituting the main bottleneck. Initial experiences for rectal cancer showed that this workflow can take up to 45 min per fraction, with 20–30 min spent on manually editing contours [3]. In rectal cancer, day-to-day variation in endorectal content can impact the tumor to background contrast, making online delineation of the gross tumor volume (GTV) under time pressure more challenging. While contour propagation methods such as deformable image registration can reduce the workload, substantial interfractional variations may lead to inaccurate contours of the rectal tumor, requiring manual corrections by a clinician [4].

Deep-learning (DL) auto-segmentation methods have been explored to obtain accurate and fast contours, particularly using multi-parametric MRI for rectal GTV auto-segmentation [5], [6], [7], [8]. However, in a typical online adaptive workflow, the rectal GTV is delineated only on T2-weighted MRI [9]. Auto-segmentation of the rectal GTV using T2-weighted MRI images exclusively, have resulted in Dice Similarity Coefficients (DSC) between 0.74 and 0.87 [10], [11], [12], [13].

While promising, these auto-segmentation methods still require significant manual adjustments, limiting the potential time-gain. In particular, GTV auto-segmentation is challenging due to tumor heterogeneity and patient-specific clinical decisions. In the context of online adaptation, however, these challenges could be mitigated by taking patient-specific prior information from previous fractions into account.

Various approaches for incorporating patient-specific information have been explored for segmentation of GTV, Clinical Target Volume (CTV), and OAR across different treatment sites [14], [15], [16], [17], [18], [19]. One strategy entailed fine-tuning a pre-trained, population-based model with previous images from the same patient, and rendering it patient-specific [14], [15], [17], [18], [19]. Alternatively, in previous work, our group trained an auto-segmentation model with not only the current fraction image but also with the patient-specific segmentation on the pre-treatment planning scan as additional input channel, for segmentation of the mesorectum CTV [16]. This approach focusses the model to look at the right location, but does not take patient-specific contrast into account. Since interfraction variation in rectal content is expected to have a bigger impact on GTV auto-segmentation compared to CTV auto-segmentation, the question arises whether previous methods of providing patient-specific information only once is appropriate for propagation of patient-specific information, or if alternative methods capturing variation in rectal content might be more suitable.

In this study, we therefore aimed to explore three approaches for integrating patient-specific prior information into rectal GTV auto-segmentation for online adaptive MRI-guided RT (MRIgRT). We compared these methods to a conventional DL approach that does not incorporate patient-specific information (‘MRI_only’).

The first approach (‘MRI+prior’), similar to previous work in our group [16], trained a model with a second input channel containing the patient-specific GTV segmentation of an earlier time-point. We hypothesize that if providing information about tumor position alone is sufficient, this model will suffice for consistent rectal GTV segmentation. For the second approach (‘patient-specific fine-tuning on fraction 1′), the benchmark MRI_only model was made patient-specific, by retraining it on a GTV segmentation from a single earlier time-point. Here we hypothesize that fine-tuning for each patient not only localizes the tumor but also provides supplementary patient-specific information to account for interpatient rectal content and anatomical variation. For the third approach (‘cumulative patient-specific fine-tuning’), patient-specific fine-tuning was performed on all earlier time-points, aggregating information from all available previous fractions. Here we hypothesize that providing not only information about rectal content once but also information on day-to-day intrapatient rectal content variation may enhance the model's robustness to interfraction variability in later fractions.

## 2 Materials and methods

### 2.1 Data

A total of 49 patients with intermediate risk or locally advanced rectal cancer treated in our institution on the 1.5 T MR-Linac (Unity, Elekta AB, Stockholm) between 2018 and 2022 were included. All patients participated in the Momentum registration study (NCT04075305) with ethics approval from the Medical Ethics Committee of the Netherlands Cancer Institute and provided informed consent for retrospective data use. Treatment involved short course RT in 5 fractions of 5 Gy with an adapt to shape (ATS) procedure. For each patient, 3D T2-weighted images acquired for adaptation (MRI_adapt_) and position verification after treatment (MRI_post_) were used from 5 fractions. For two patients, the final fraction was performed at the conventional linac, resulting in a total of 243 images. All images were acquired with field of view (FOV): 400 × 448 × 249 mm^3^, repetition time (TR): 1300  ms, echo time (TE): 128  ms. Voxel size was 1.2 × 1.2 × 1.2 mm^3^ for MRI_adapt_ and 1.2 × 1.2 × 2.4 mm^3^ for MRI_post_. As GTV delineations on the planning CT were not available, delineations of the first fraction served as a surrogate to propagate prior patient-specific information to subsequent fractions.

The gross tumor volume of the primary tumor (GTV) was delineated retrospectively by two radiation technology therapists (RTTs) using Monaco v5.40.01 (Elekta, Stockholm, Sweden). All images of one patient were delineated and adjusted by the same RTT. On MRI_adapt_ of the first fraction, GTV was delineated using pre-treatment endoscopic reports and diagnostic MRI, and further optimized based on expert input of 2 radiation oncologists and a radiologist. The delineation was then rigidly copied to fractions 2 to 5 and manually adjusted. For 10 patients, delineations on MRI_adapt_ were rigidly copied to MRI_post_ of the same fraction and manually adjusted to evaluate post-treatment coverage.

### 2.2 Model architecture and training

#### 2.2.1 Model architecture

The no-new-net (nnU-net) framework [20] was used to train 3D models for rectal GTV segmentation, with automatic data augmentation and default settings. Details are provided in Table S1.1. The dataset was split on a patient level into a training set consisting of 29 patients (144 images), a validation set of 10 patients (50 images) and a test set of 10 patients (49 images). Only patients with a GTV delineation on MRI_post_ were included in the test set for post-treatment coverage evaluation, ensuring an equal distribution of cases assessed by all observers and levels of complexity of tumors concerning rectal contrast variability. For the training and validation set random splitting was performed. Post-processing was performed by selecting the largest segmented volume to remove small disjoint voxels across all approaches. Automated contours were not manually corrected.

#### 2.2.2 Model training

**Benchmark: MRI_only**

A population model was trained as benchmark. The model used as input 3D T2-weighted images of fractions 2 to 5 to segment the rectal GTV (Figure S2.1).

**MRI+prior**

In this approach, a model was trained with a second input channel containing the segmentation from fraction 1 rigidly registered to fractions 2–5 (Figure S2.2). This strategy aimed to replicate the clinical workflow, where decisions made during delineation for planning are consistently propagated to online treatment fractions. Fraction 1 images were automatically rigidly registered to the current fraction images using the Elastix toolbox [21], [22] and structures were propagated accordingly. For prediction on the test set, the trained model was applied to images of fraction 2–5 along with corresponding patient-specific propagated segmentations from fraction 1.

**Patient-specific fine-tuning on fraction 1 (PSF_1)**

To evaluate if adding tumor location and patient-specific contrast information once enhances model performance, patient-specific models were made by retraining the MRI_only benchmark model on fraction 1 of each patient of the test set (Figure S2.3). Following Smolders et al [17], all layers were optimized. Training for fine-tuning was stopped after 5 epochs, as further iterations showed negligible changes in validation set loss. Patient-specific models were derived on fraction 1 and applied for predictions on fractions 2 through 5 for each patient using images and segmentations of that fraction as input.

**Cumulative patient-specific fine-tuning (PSF_cumulative)**

To assess whether additional information about intrapatient variation is beneficial, the MRI_only model was retrained for each patient in the test set on images and segmentations from all previous fractions (Figure 2.4), obtaining one model per patient and fraction. Training for fine-tuning was stopped after 5 epochs. Patient-specific models were derived on each fraction and applied for predictions on the next fraction for each patient using images and segmentations of that fraction as input.

### 2.3 Performance evaluation

For each patient in the test set, segmentations from each method were compared to the manual GTV segmentations of fractions 2–5 using the DSC, 95th percentile of Hausdorff distance (95HD), Surface Dice with a tolerance of 2.0 mm (SD_2mm_) [23], [24] and the Added Path Length (APL) [25]. Surface Dice and APL correlate well with manual editing time [25], whereas 95HD estimates the maximum error. APL was calculated, using in-house code, as the surface voxels of the binary mask (using the resolution of MRI_adapt_ (1.2 × 1.2 × 1.2 mm*^3^*) requiring editing and values are reported as the number of voxels similar to Kiser et al [26]. Similar to Doolan et al [27], no additional tolerance was used so that all edits are assessed. The implicit tolerance is, at most, equal to the voxel size. Other metrics were calculated using the Python package surface distance (https://github.com/deepmind/surface-distance). For every metric, a Wilcoxon signed rank test was conducted for pairwise comparison of the different approaches (MRI_only vs MRI+prior; MRI_only vs PSF_1, MRI_only vs PSF_cumulative, MRI+prior vs PSF_1, MRI+prior vs PSF_cumulative). To determine whether accumulation of information was required to account for intrapatient variation, we compared model performance on fraction 3–5 to fraction 2, using a Wilcoxon signed rank test. An α of <0.05 was considered statistically significant and we corrected for multiple testing using Bonferroni correction. The SciPy Python package (version 1.5.4) was used for statistical analysis.

**Evaluation of the effect of segmentation approaches on post-treatment volumetric coverage**

In addition to pairwise geometric comparison, we evaluated the volumetric coverage of the PTV derived from the GTV on MRI_adapt_ and retrospectively manually delineated GTV on MRI_post_. This evaluation was done for each method for patients in the test set using methods from previous work [16], [28]. For each method separately, the automatically segmented GTV was expanded in steps of 0.5 mm to a PTV. The expansion in LR direction was 2.5x smaller compared to all other directions, as motivated in earlier work on GTV intrafraction motion [29]. For each patient, the volumetric overlap with the GTV on MRI_post_ was averaged over all fractions.

Subsequently, the coverage reached by at least 90% of the patients was determined for each expansion. As a reference, post-treatment coverage using the retrospectively manually delineated contours on MRI_adapt_ was also determined.

## 3 Results

Patient and tumor characteristics of our cohort are described in Table S3.1.

### 3.1 Performance

**Fig. 1:**
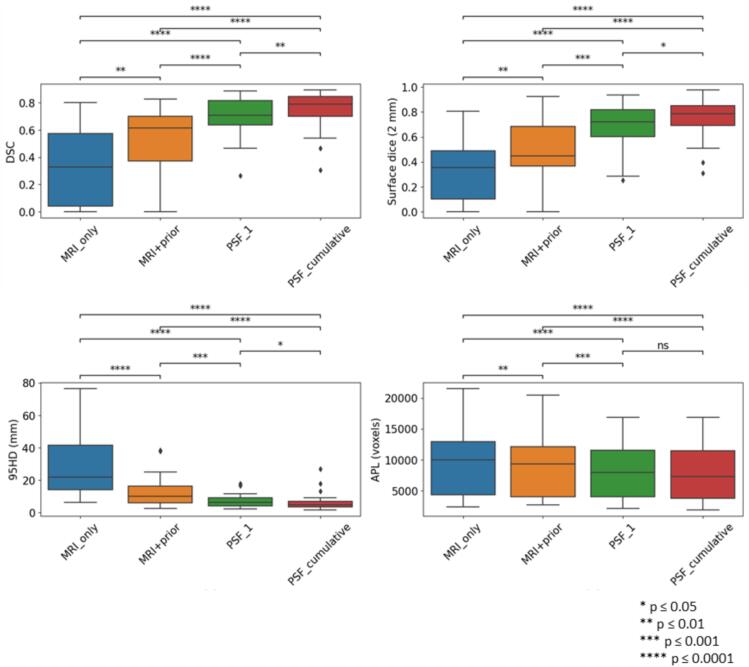
Performance of the MRI_only, MRI+prior, Patient-specific fine-tuning on fraction 1 (PSF_1) and patient-specific fine-tuning by aggregating data from previous time-points (PSF_cumulative) for the patients in the test set.

The performance of the various segmentation approaches is shown in Fig. 1. Results before post-processing are provided in Figure S5.1 in the supplementary materials. All patient-specific methods outperformed the MRI_only approach. Median 95HD were 22.0 (range: 6.1–76.6) mm for the MRI_only segmentation approach, 9.9 (range: 2.5–38.2) mm for the MRI+prior approach, 6.4 (range: 2.4–17.8) mm for PSF_1 and 4.8 (range: 1.7–26.9) mm for PSF_cumulative. Visual inspection revealed that in 10 fractions, the MRI_only segmentations did not overlap with the manual GTV. In addition, for 2 patients, it failed to provide a prediction in 1 fraction. When adding prior information as a second input channel (MRI+prior), the model was able to locate the tumor but generally struggled with determining tumor borders. PSF_1 improved border detection although for 2 patients with relative large variation in rectal filling in later fractions, the model undersegmented the tumor at fraction 4 and 5. PSF_cumulative consistently outperformed all approaches. Pairwise comparison showed that the performance between approaches was statistically significant (p < 0.05) for SD_2mm_ and 95HD. For APL, there was no significant difference between PSF_1 and PSF_cumulative. For one patient, there was a discrepancy between the manual contours of fractions 3 and the previous fractions due to large tumor deformation as a result of gas and faeces and insufficient tumor-to-background contrast relative to fraction 1. PSF_cumulative therefore had difficulty segmenting the tumor accurately, resulting in 95HD of 17.8, 26.9 and 13.2 mm for fractions 2, 3 and 4 respectively. Other approaches also encountered difficulty in segmenting the tumor for this patient with the MRI_only approach failing to segment entirely for fraction 3.

**Fig. 2:**
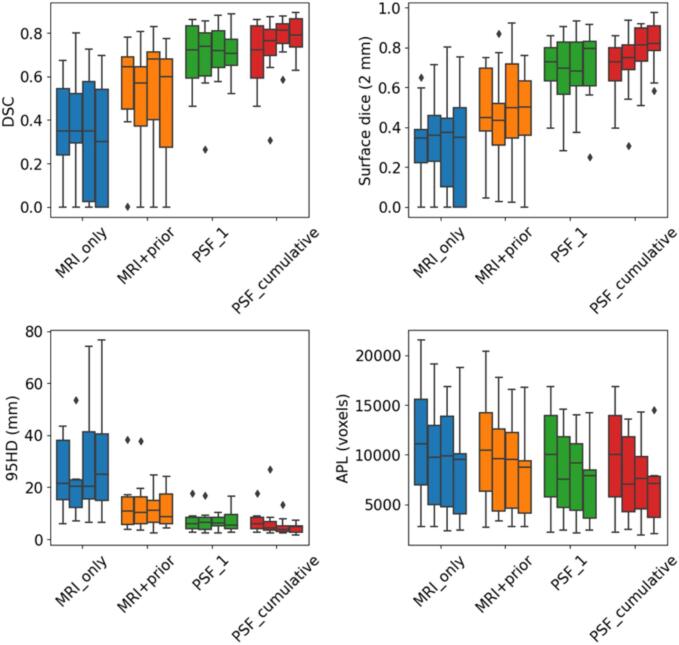
Performance of the MRI_only, MRI+prior, Patient-specific fine-tuning on fraction 1 (PSF_1) and patient-specific fine-tuning by aggregating data from previous time-points (PSF_cumulative) for each fraction of the patients in the test set. The 4 boxes per approach represent the performance in fractions 2 to 5.

We additionally evaluated the performance of the segmentation approaches relative to Fraction 2 to determine whether accumulation of information was required to account for intrapatient rectal content variation (Fig. 2). With the exception of PSF_cumulative, there were no discernible differences in performance across fractions. PSF_cumulative performed better from fraction 4 onwards compared to fraction 2 (p = 0.014), with no significant further improvement from fraction 4 to 5 (p = 0.12).

**Fig. 3:**
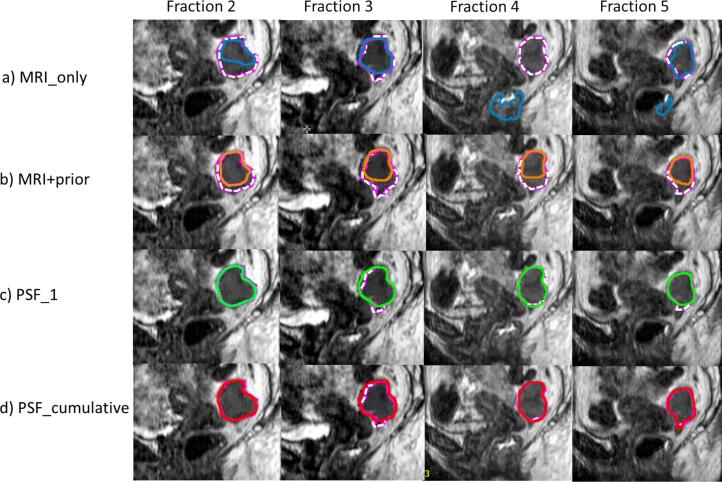
Comparison of the auto-segmentation results of the a. MRI_only (blue), b. MRI+prior (orange), c. patient-specific fine-tuning on fraction 1 (PSF_1; green) and d. patient-specific fine-tuning by aggregating data from previous time-points (PSF_cumulative; red) approach for fractions 2–5 (left to right) to manual segmentations (dashed lines). (For interpretation of the references to colour in this figure legend, the reader is referred to the web version of this article.)

Fig. 3 shows examples of the segmentations obtained from all methods applied to fractions 2–5 of a single patient. Notably, the MRI_only approach yielded varied predictions across the fractions and segmented additional areas in the lower part of the mesorectum in fractions 4 and 5. The MRI+prior model consistently undersegmented the tumor caudally in all fractions of this patient possibly due to insufficient tumor-to-background contrast, whereas with the fine-tuned approaches the predictions closely aligned with the manual segmentations.

**Evaluation of the effect of segmentation approaches on post-treatment coverage**

**Fig. 4:**
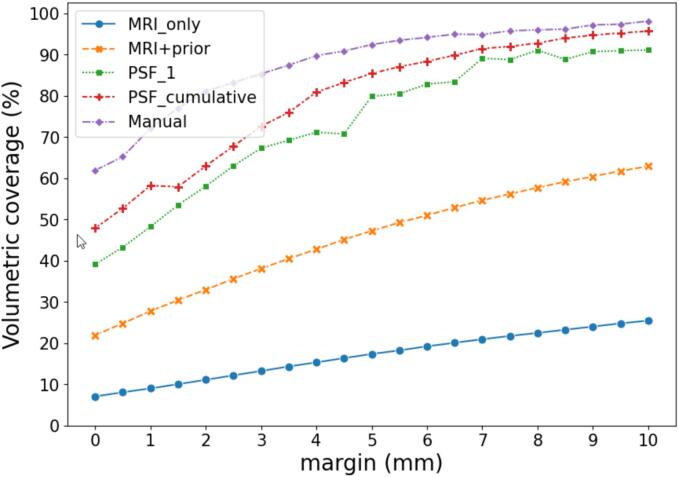
Post-treatment volumetric coverage for 90% of the population when using segmentations obtained from the MRI_only approach (blue), MRI+prior approach (orange), PSF_1 (green), PSF_cumulative (red) and manual segmentations (purple). (For interpretation of the references to colour in this figure legend, the reader is referred to the web version of this article.)

The post-treatment GTV coverage for MRI_only, MRI+prior, PSF_1, PSF_cumulative and the manual segmentations were compared (Fig. 4). In terms of coverage, similar to the geometric evaluation, increasing performance was observed by using the different segmentation approaches. If a PTV margin of at least 5.0 mm (2.5 mm LR) as suggested by Kensen et al [29] would be used, a relative volumetric coverage of 17, 47, 80, 85 and 92% would be reached in 90% of the population for the MRI_only, MRI+prior, PSF_1, PSF_cumulative and manual approach, respectively.

## 4 Discussion

In this study, we assessed various methods of incorporating patient-specific information for the auto-segmentation of rectal tumors in the context of online adaptive MRIgRT. Specifically, we compared a MRI+prior segmentation approach, patient-specific fine-tuning on the first fraction (PSF_1), and patient-specific fine-tuned models on all earlier treatment fractions (PSF_cumulative) against a MRI_only approach. Our findings demonstrate that utilizing an auto-segmentation approach based on accumulated anatomical information from previous fraction scans of the same patient yielded the most accurate results for segmenting rectal tumors across all treatment fractions.

The performance of the MRI_only approach was generally inferior compared to the other approaches. In certain instances, the model classified non-tumor areas within the mesorectum as tumor. Furthermore, the model encountered challenges in delineating tumor borders, leading to both under- and oversegmentation. This difficulty may stem from variations in tumor location and tumor-to-background contrast between the images utilized in the training and test sets. Additionally, the model struggled to deliver consistent predictions for different fractions of a patient due to interfractional variability in rectal filling.

When comparing our result for the MRI_only approach to literature, studies generally performed better than our study with median DSC between 0.74 and 0.87 [10], [11], [12], [13]. This may be due to the differences in cohort sizes and the use of different approaches to first detect the tumor to improve segmentation performance. In our study, localization of the GTV was done implicitly by using the patient-specific prior, allowing a substantial improvement with much less patients.

With regards to adding patient-specific information by means of a prior segmentation, we observed a significant difference in the performance when compared to patient-specific fine-tuning on the first fraction (PSF_1). This suggests that solely providing information on the tumor’s location is insufficient to accurately segment rectal GTVs. In previous work within our group [16], an MRI+prior approach adding a prior voxel-wise probability map of the mesorectum as a second input channel was utilized to segment the mesorectum CTV resulting in a median DSC of 0.89. Here, supplementing the patient-specific information with population-based information on mesorectum shape variation accommodated the day-to-day variation in mesorectum shape. However, the GTV is not an anatomical structure and varies depending on the stage, shape and location. This is likely the reason why patient-specific fine-tuning significantly improves results compared to the MRI+prior approach.

Previous research on patient-specific fine-tuning focused on similar approaches for segmenting GTVs and OARs for different treatment sites. Fransson et al. [14] utilized a model fine-tuned on fraction 1 images for prostate cancer CTV and OAR segmentation, observing significant performance variation in rectum segmentation due to interfraction variability in rectal volume. Elmahdy et al. [15] also observed variation in rectum segmentation performance when updating patient-specific models of each previous fraction. Kawula et al. [30] also generated patient specific models for OAR delineation and showed that model performance improved. Similar to previous studies, they found that if there was a large difference in rectal filling on the day of treatment as compared to the planning image, the accuracy of the fine-tuned model decreased. These findings imply that for rectum prior information should capture possible variation in rectal filling to accurately segment contours. Comparing the performance of each approach to Fraction 2, significant improvement was noted at Fraction 4 with PSF_cumulative. This suggests that there is intrapatient variation in rectal contents and accumulating anatomical information increases likelihood of obtaining representative images for the current fraction. The incremental addition of information from fraction 4 only marginally improved performance on fraction 5, suggesting that the additional anatomical information in our dataset may not significantly differ from previous time-points.

In addition to geometrical and distance based metrics, we reported the post-treatment volumetric coverage as a surrogate for dosimetric coverage when using the auto-segmentation approaches and manual segmentations. This showed that PSF_cumulative had similar performance compared to the manual segmentations. This analysis is however impacted by interobserver variation (IOV) in the auto-segmentation, which can be up to 2 mm for the GTV [24]. Since MRI_post_ is delineated based on the MRI_adapt_ delineation, the effect of the IOV is expected to be smaller compared to the case where the delineation is made independently, as with auto-segmentations. For an unbiased comparison, the tumor should ideally be delineated independently on MRI_post_.

We demonstrated the feasibility of patient-specific fine-tuning for GTV auto-segmentation, though the model was not optimized for fast inference. Clinical use would require allocation of staff to perform offline retraining after each fraction to obtain a fine-tuned model for the next fraction. Additionally, it would require logistic and regulatory considerations including the availability of appropriate hardware for fast inference, and MDR compliant quality assurance. While this may not be feasible for most hospitals now, commercial adoption by vendors could make patient-specific retraining a standard practice, considering its clinical potential in reducing contouring time.

In conclusion, this study compared various methods of incorporating patient-specific information for the DL-based auto-segmentation of rectal tumors in the context of online adaptive MRIgRT. Our findings indicate that patient-specific fine-tuning using images and segmentations from all previous fractions yields superior contour quality in the presence of interfractional variations in rectal content, compared to other approaches.

## Declaration of Generative AI and AI-assisted technologies in the writing process

During the preparation of this work, the author(s) used ChatGPT (https://chat.openai.com) to enforce the word count limit. After using this tool/service, the author(s) reviewed and edited the content as needed and take(s) full responsibility for the content of the publication.

## CRediT authorship contribution statement

**Chavelli M. Kensen:** Formal analysis, Investigation, Writing – original draft, Visualization. **Rita Simões:** Conceptualization, Methodology, Writing – review & editing. **Anja Betgen:** Data curation, Writing – review & editing. **Lisa Wiersema:** Data curation, Writing – review & editing. **Doenja M.J. Lambregts:** Data curation, Writing – review & editing. **Femke P. Peters:** Data curation, Writing – review & editing. **Corrie A.M. Marijnen:** Data curation, Writing – review & editing. **Uulke A. van der Heide:** Conceptualization, Methodology, Supervision, Writing – original draft. **Tomas M. Janssen:** Conceptualization, Methodology, Supervision, Writing – original draft.

## Declaration of competing interest

The authors declare the following financial interests/personal relationships which may be considered as potential competing interests: UAH is an Editorial Board Member for *Physics and Imaging in Radiation Oncology* and was not involved in the editorial review or the decision to publish this article. UAH, CAMM and TMJ report institutional funding by Elekta AB. UAH reports research support by Elekta AB and research support by Philips Healthcare. The remaining authors have no competing financial interests or personal relationships that could have appeared to influence the work reported in this paper.
