# Supplementary material for: Incorporating patient-specific information for the development of rectal tumor auto-segmentation models for online adaptive magnetic resonance Image-guided radiotherapy
Source: Phys Imaging Radiat Oncol. 2024 Sep 16;32:100648. doi: 10.1016/j.phro.2024.100648 (PMC11421252; doi:10.1016/j.phro.2024.100648)
Supplement: Supplementary Data 1 [file mmc1.docx]

Supplementary Materials

This document contains supplementary information for the manuscript “Incorporating patient-specific information for the development of rectal tumor auto-segmentation models for online adaptive Magnetic Resonance Image-guided radiotherapy”

# **1 Model architecture & training details**

Table S1.1: Training details.

|  | MRI_only & MRI+prior | PSF_1 & PSF_cumulative |
| --- | --- | --- |
| Optimizer | Stochastic Gradient Descent | Stochastic Gradient Descent |
| Loss function | Dice & cross entropy | Dice & cross entropy |
| Initial learning rate | 0.01 | 0.01 |
| Learning rate scheduler | Poly | Poly |
| Epochs | 1000 | 5 |
| Training times | 12h | 3 min 30 seconds |
| Inference time for a single images | 1 min 30 seconds | 1 min 30 seconds |
| Early stopping | Based on the best performing weight configuration on the validation set | - |
| Hardware | 4 NVIDIA A100 GPUs | 4 NVIDIA A100 GPUs |

**2 Visual representation of methods**
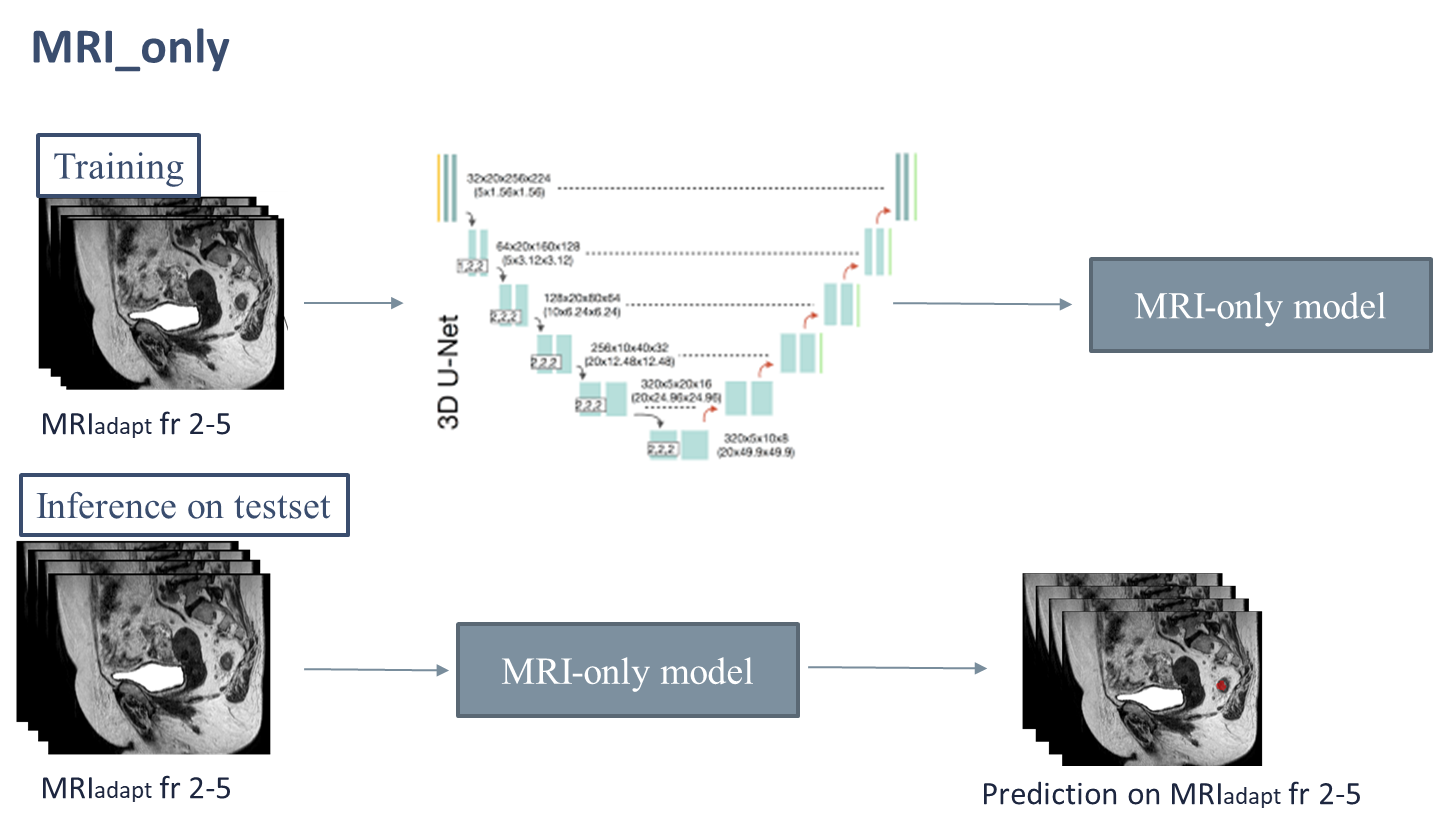


Figure S2.1: Overview of training and inference for the MRI-only model, which served as a benchmark. The model was trained on fraction 2-5 MRI and then used for inference on patients in test set.


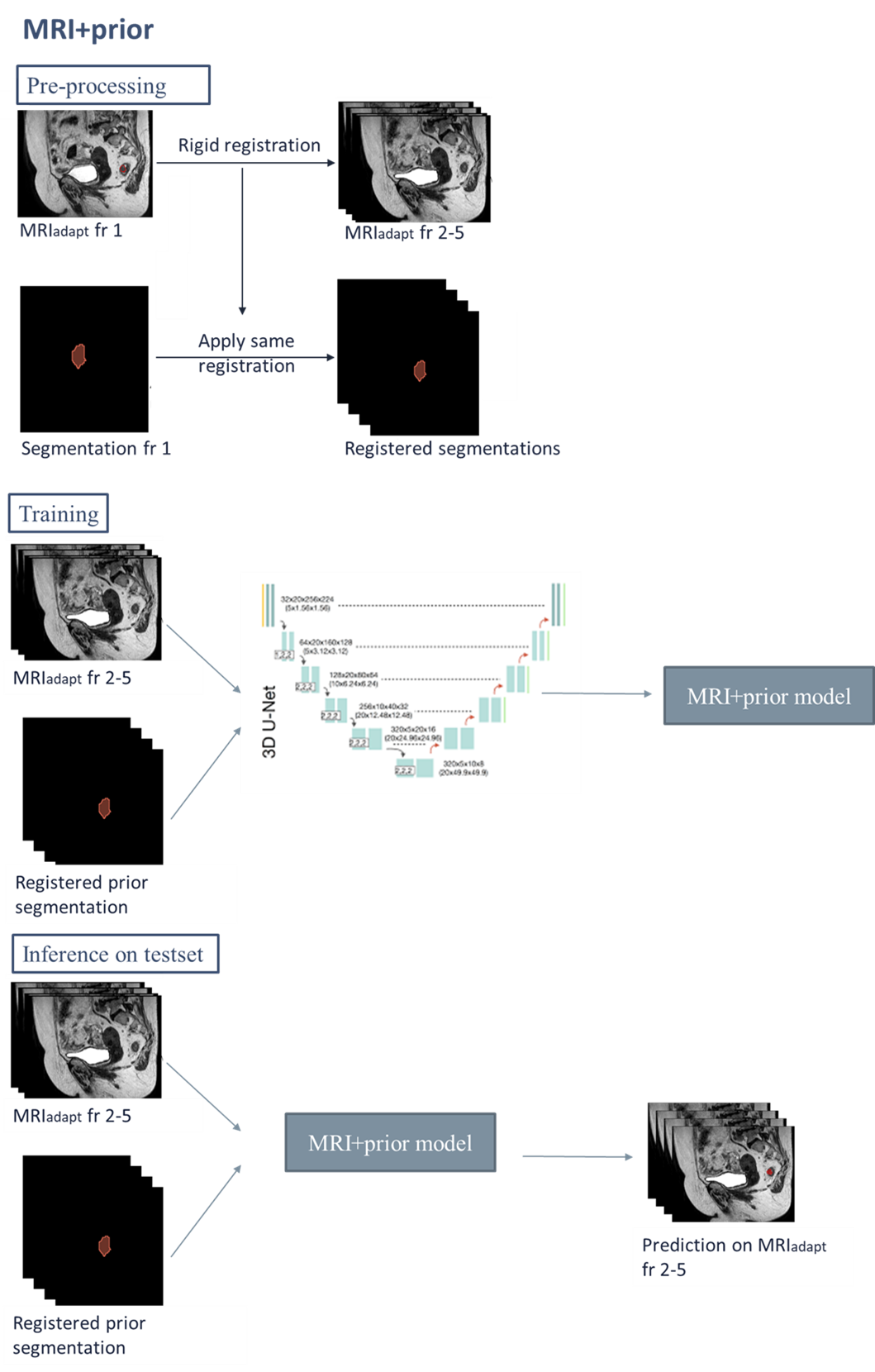


Figure S2.2: Overview of preprocessing, training and inference for the MRI+prior model. Here the model is trained on fraction 2-5 MRIs and the first fraction segmentation as a second input.


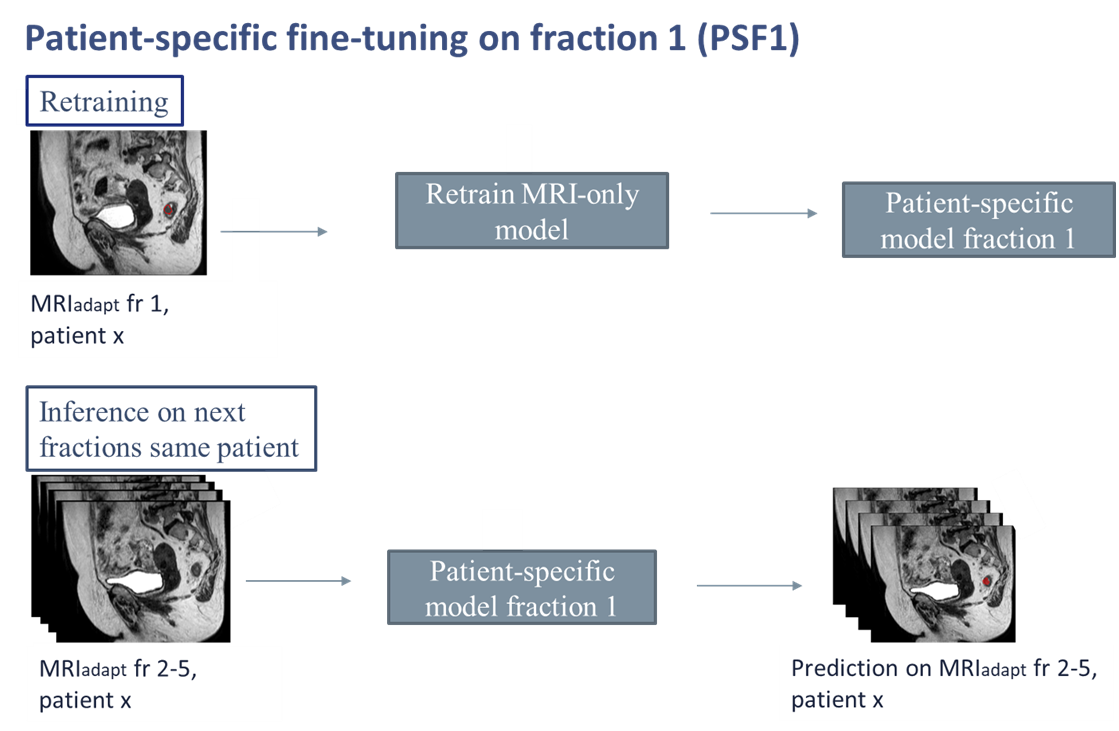


Figure S2.3: Overview of training and inference for Patient-specific fine-tuning on fraction 1 (PSF_1). The MRI-only model is retrained on MRIadapt fraction 1 to obtain a patient-specific model for fraction 1, which is in turn used for prediction on fraction 2-5.


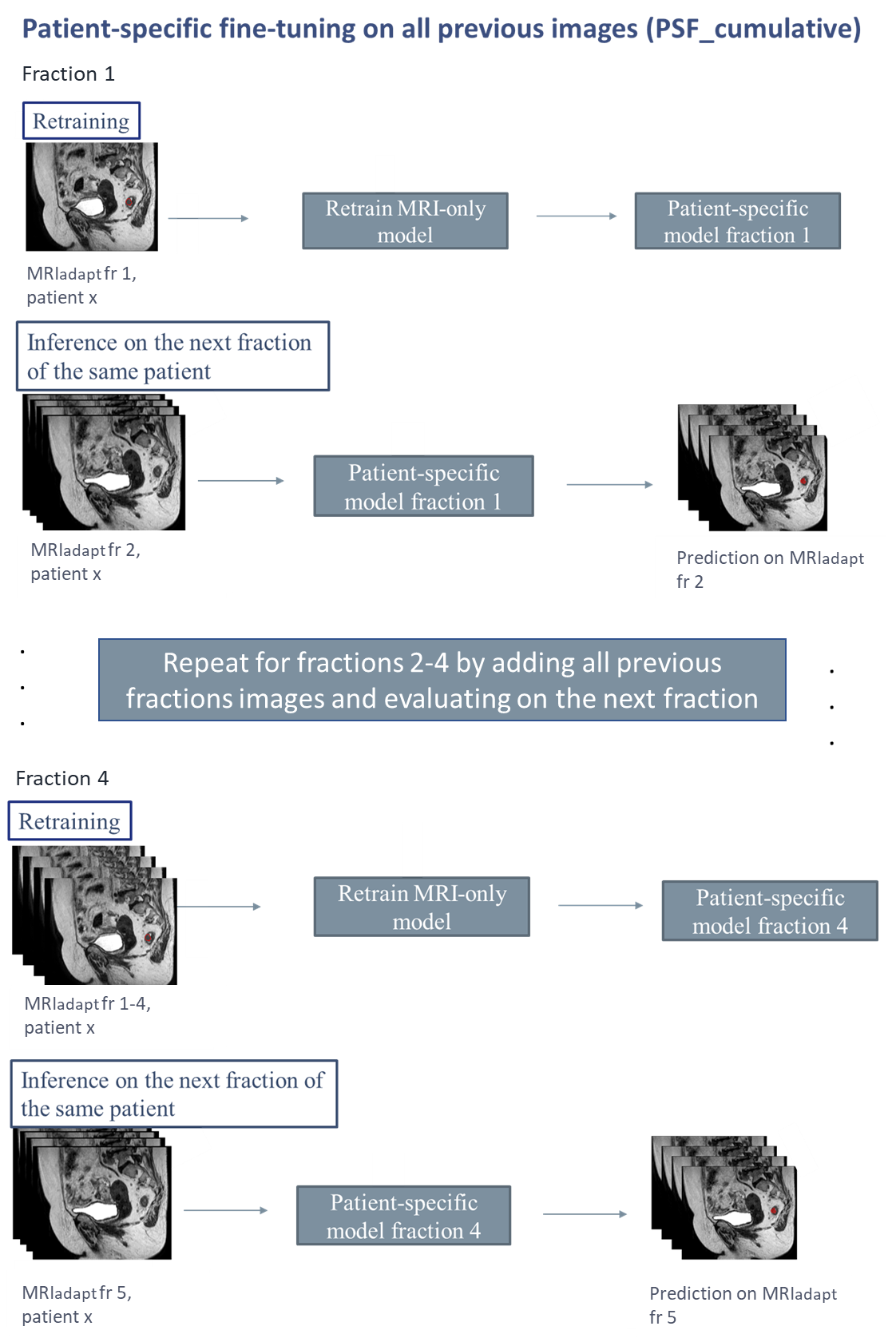


Figure S2.4: Overview of training and inference for cumulative Patient-specific fine-tuning (PSF_cumulative). For each patient in the test set, the MRI-only model is retrained on all previous fraction images to obtain a patient-specific model for each fraction, which is in turn used for predictions on the next fraction.

# **3 Patient characteristics**

Table S3.1: Patient characteristics

| Patient characteristics | N=49 (%) |
| --- | --- |
| Age in years (median; range) | 61; 34-81 |
| Sex |  |
| Male | 34 (69.4) |
| Female | 15 (30.6) |
| Tumor stage |  |
| cT1-2 | 10 (20.4) |
| cT3 | 35 (71.4) |
| cT4 | 4 (8.2) |
| Nodal stage |  |
| N0 | 17 (34.7) |
| N1 | 25 (51.0) |
| N2 | 7 (14.3) |
| Tumor location (distance to anorectal junction) |  |
| Lower rectum (0 to ≤ 5 cm) | 34 (69.4) |
| Mid rectum (> 5 to 10 cm) | 12 (24.5) |
| Upper rectum ( > 10 cm) | 3 (6.1) |

# **4 Model performance on validation set**


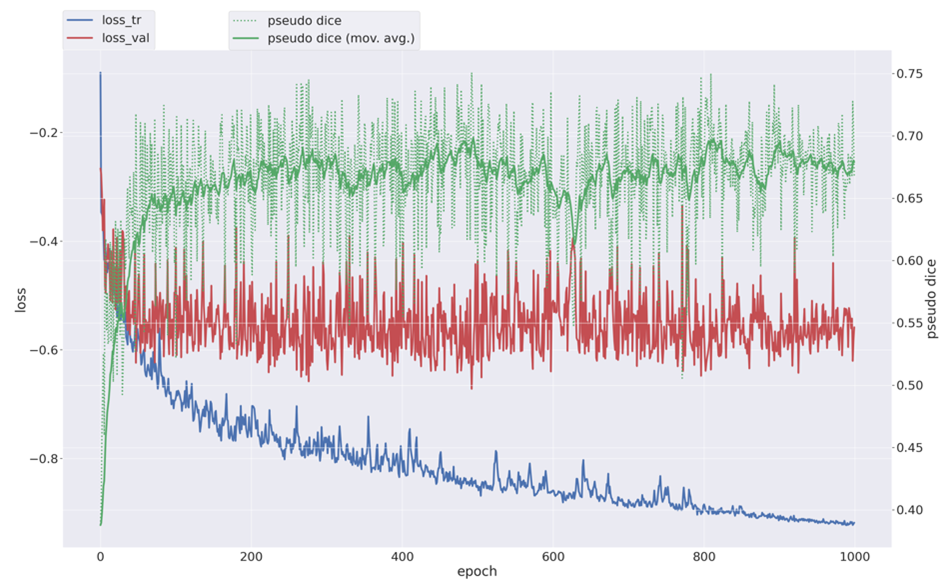


Figure S4.1: Performance of MRI-only model during training. The training loss (blue), validation loss (loss) & pseudo dice (green) curves are depicted for the training process.


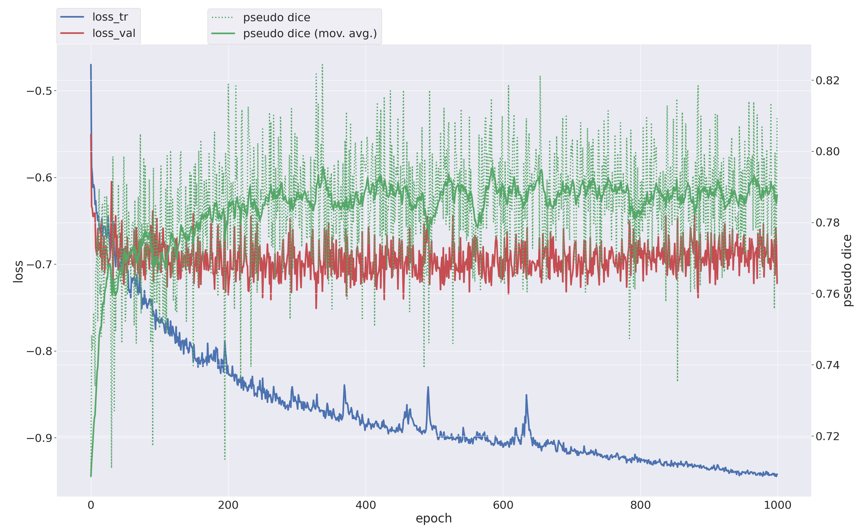


Figure S4.2: Performance of MRI+prior model during training. The training loss (blue), validation loss (loss) & pseudo dice (green) curves are depicted for the training process.


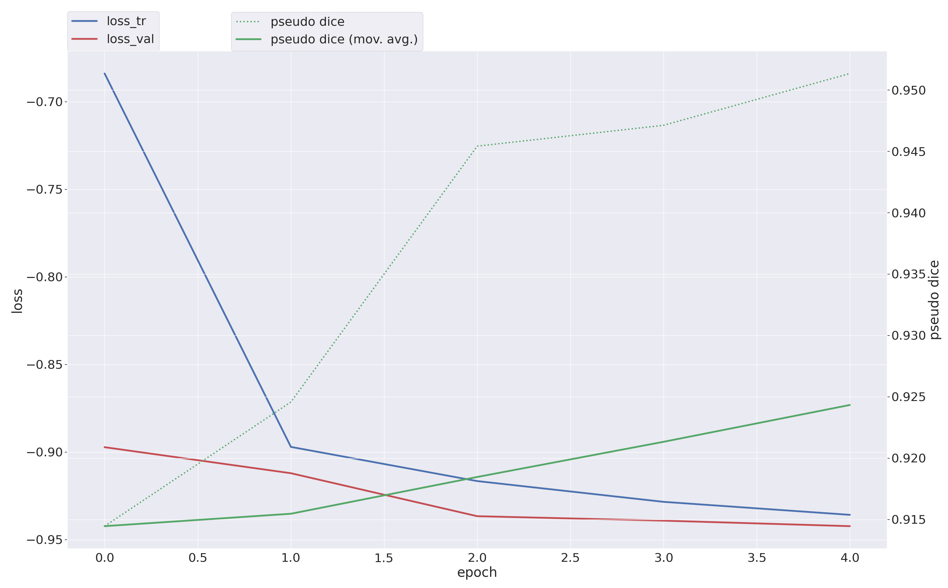


Figure S4.3: Performance of the patient-specific model for fraction 1 of one patient in the test set during training. The training loss (blue), validation loss (loss) & pseudo dice (green) curves are depicted for the training process. These curves are representative for PSF_1 and PSF_cumulative of fraction 1.

# **5 Model performance on test set before post-processing**


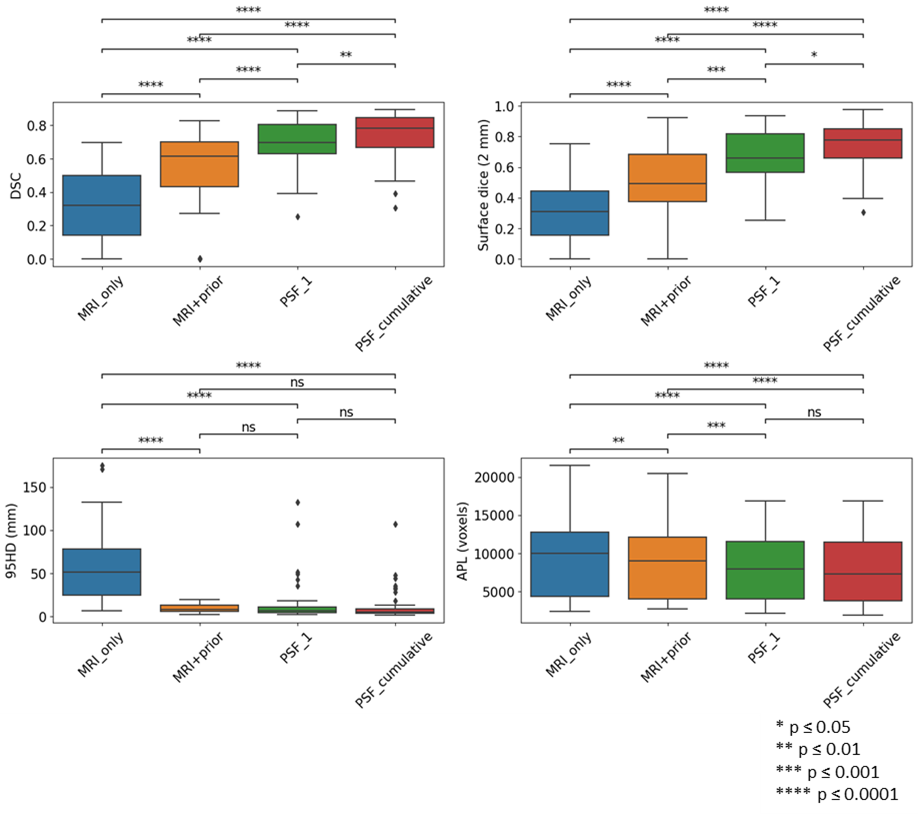


Figure S5.1: Performance of the different approaches for the patients in the test set before post-processing.
